# Effect of Isothermal Temperature on Growth Behavior of Nanostructured Bainite in Laser Cladded Coatings

**DOI:** 10.3390/ma10070800

**Published:** 2017-07-14

**Authors:** Yanbing Guo, Chengwu Yao, Kai Feng, Zhuguo Li, Paul K. Chu, Yixiong Wu

**Affiliations:** 1 School of Mechanical Engineering, Shanghai Dianji University, Shanghai 201306, China; yanbingg1984@126.com; 2 Shanghai Key Lab of Materials Laser Processing and Modification, School of Materials Science and Engineering, Shanghai Jiao Tong University, Shanghai 200240, China; yaochwu@sjtu.edu.cn (C.Y.); fengkai@sjtu.edu.cn (K.F.); yxwu@sjtu.edu.cn (Y.W.); 3 Department of Physics and Materials Science, City University of Hong Kong, Tat Chee Avenue, Kowloon 999077, Hong Kong, China; paul.chu@cityu.edu.hk

**Keywords:** nanostructured bainite, sheaves, growth behavior, laser cladded coating, orientation relationship

## Abstract

The growth and propagation behavior of austenite-to-bainite isothermal transformation in laser-cladded, Si-rich, and Fe-based coatings is investigated. The crystallographic features, orientation relationship at different isothermal temperatures, and the morphology of the nanostructured bainite are determined. The Nishiyama-Wassermann type orientation relationship is observed at a high temperature and at a low temperature, and mixed Nishiyama-Wassermann and Kurdjumov-Sach mechanisms are seen. The growth direction is investigated by the partial dislocation theory and an extrapolated model based on the repeated formation of lenticular-shaped subunits and pile-up along the close-packed directions of the close-packed planes. The variants of the bainite growth directions would be more selective at the high transformation temperature.

## 1. Introduction

A new generation of carbide-free nanostructured bainitic steel has been developed [1,2,3]. The large concentrations of carbon and silicon in the steel lead to slow bainitic transformation at a low temperature (200 °C) and an ultra-fine nanoscaled structure composed of bainitic ferrite lathes and retained austenite films between these lathes [2,4,5]. Nanostructured bainitic steel, which possesses ultra-high tensile strength (>2 GPa) and a toughness of 30 MPa·m^1/2^, has large industrial potential, but has the drawback of having a slow transformation process that usually requires several days. The transformation process can be accelerated by increasing the driving force of the bainitic transformation by adding Co and Al or by refining the austenite grain size to increase the initial nucleation sites [6]. For instance, laser cladding with a large cooling rate has been used to fabricate fine primary austenite grains [7] and aluminum and cobalt have been introduced to enhance the austenite-ferrite transformation [6].

Austenite “blocks” are distributed at the interdendritic boundaries in laser-cladded coatings due to microsegregation during solidification [8]. Segregation at the interdendritic boundaries affects the kenitics of bainitic transformation and, as a result, the morphology and distribution of the nanostructured bainite in the cladded coatings vary with the isothermal temperature [9]. The microstructural characteristics such as the crystallographic arrangement and the mutual crystallographic relationship between the bainite sheaves can be utilized to elucidate the nucleation and propagation processes and, furthermore, the mechanical properties are impacted by these microstrcutural characteristics [10,11,12,13]. Although the morphology and crystallography of nanostructured bainite have been studied [14,15], the evolution of the bainitic morphology and the bainitic microstructure disribution at different temperature has seldom been studied. As previously reported [15,16], the relationship between nanobainitic ferrite and austenite is Nishiyama-Wassermann (N-W) instead of Kuidjumov-Sachs (K-S), and there are 24 variants in the K-S relationship and 12 variants in the N-W relationship between bainitic ferrite and austenite [17,18]. In this work, the growth and propagation behavior of the bainitic transformation at different isothermal temperatures and the variants of the crystal orientations between the γ (austenite) and α (bainitic ferrite) phases are studied. A model based on the morphology and distribution of the nanobainitic sheaves and orientation relationship of the different phases is postulated and described.

## 2. Materials and Experimental Procedures

The precursor materials used in laser cladding were 75–250 μm spherical powders fabricated by the plasma rotation electrode process. The composition of the powders was Fe-0.81 C-1.55 Si-1.96 Mn-1.13 Cr-0.32 Mo-1.53 Co-0.93 Al (wt. %). The substrate was mild steel [Fe-0.14 C-0.22 Si-0.58 Mn (wt. %)] with dimensions of 160 mm × 15 mm × 12 mm. The substrate was polished and then cleaned with acetone and alcohol prior to laser cladding. The cladding system (Rofin DL-035Q, Plymouth, MI, USA) consisted of a 3.5 kW diode laser, a five-axis robotic laser scanning control, and a powder coaxial nozzle feeding system with a gas shielding device to protect the coatings from oxidation. The argon was employed as shielding gas with a flow rate of 15 L/min. The spot size of the laser at the focal length (165 mm) is 2.5 mm × 3.5 mm.

The substrate was pre-heated (by using the electronic hot plate, CT-946A) above the corresponding isothermal temperature before laser cladding and the sample was transferred to a furnace for isothermal heat treatment after laser cladding. During sample transferring, a surface digital thermometer (Anritsu HA-100K, Morgan Hill, CA, USA) was used to monitor the temperature of the specimens to make sure that the temperature was not below the isothermal temperature. The specimens were quenched at ambient temperature after a certain holding time. The important instrumental parameters in laser cladding and subsequent isothermal heat treatments are listed in Table 1. Optimized laser power of 2.2, 2.3, 2.4, or 2.5 kW was used for the cladded samples, which were preheated to 370, 320, 270, and 220 °C, respectively.

After cladding and heat treatment, the specimens were sectioned transversely, polished by a conventional metallographic technique, and etched by 4% nital to reveal the microstructure. The microstructure in the central area of the coatings was studied using field-emission scanning electron microscopy (NOVA NanoSEM 230, FEI, Hillsboro, OR, USA). The foils for transmission electron microscopy (TEM) were thinned to a thickness of 80 μm with emery paper and electropolished at −30 °C in 5% perchloric acid/95% ethanol at 40 V. The thin foils were examined by transmission electron microscopy (TEM JEOL JEM-2100F, Tokyo, Japan) at 200 kV. The specimens for electron backscattered diffraction (EBSD) were prepared by mechanical grinding and vibration-polishing with a silica slurry. EBSD was performed at 20 kV, with a tilt angle of 70° and a step size of 0.1 μm.

## 3. Results and Discussion

### 3.1. Characteristics of the Microstructure of Nanobainite

**Figure 1:**
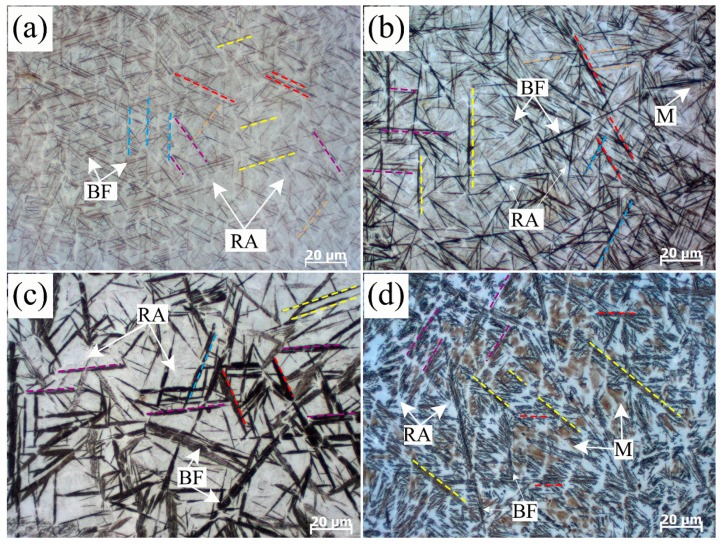
Optical micrographs of the nanobaninitc coatings after different isothermal processing at 200 °C for 4 h (**a**); 250 °C for 2 h (**b**); 300 °C for 2 h (**c**); and 350 °C for 3 h (**d**).

Optical microstructures revealed by etching are illustrated in Figure 1a and show the structure formed by the isothermal transformation at 200 °C for 4 h in the coating alloy. Black thin acicular structures have already been recognized as bainitic ferrite (BF) sheaves in the authors’ previous studies [9]. Bainitic ferrite sheaves nucleate at the austenite grain boundary initially and then grow from them into a retained austenite (RA) grain. The propagation of these sheaves grow along several particular directions (bainitic sheaves grow with the same direction are marked with the same colored dash lines, as shown in Figure 1). In the coatings decomposed at 200 °C for 4 h and 250 °C for 2 h typical acicular bainite laths form, surrounded by polygonal areas composed of RA and martensite (M) between them, as in Figure 1a,b. The 300 °C transformed sheaves present a bamboo leaves shape. The width and length of bainitic sheaves sharply increased as the transformed temperature increased from 200 °C to 300 °C. The primary transformed bainite 350 °C grows to be long sheaves, while the secondary formed bainite plates with different growing directions interlace with each other. Island-like shape bainite and martensite near the austenite grain boundaries can also be observed in Figure 1d. The scale of the sheaves of 350 °C transformed bainite is almost unchanged compared with the 300 °C transformed bainite.

**Figure 2:**
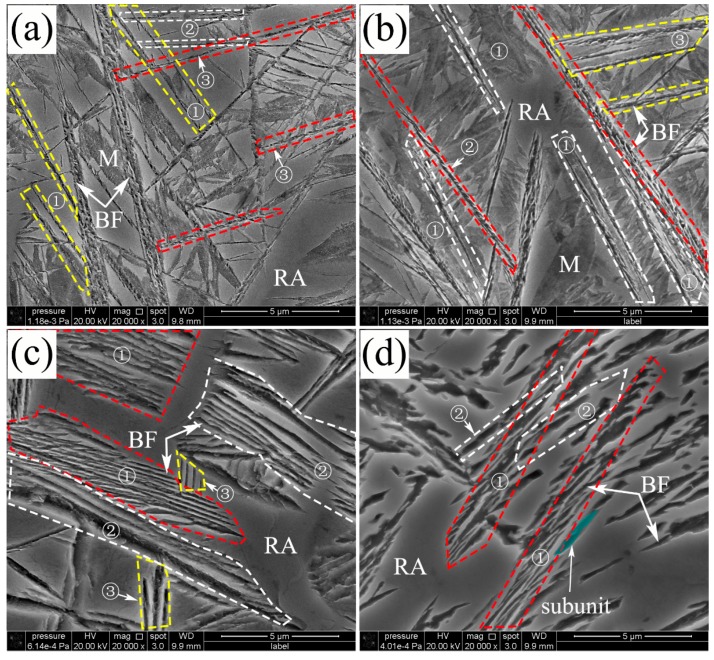
The propagation morphology and sheaves of the nanostructured bainite in the laser cladded coatings, transformed at (**a**) 200 °C; (**b**) 250 °C; (**c**) 300 °C and (**d**) 350 °C.

Additionally, FE-SEM was employed to characterize the details of the microstructure and morphology of nanostructured bainite transformed at different isothermal temperatures, as shown in Figure 2. The sheaves of bainite had a lamella morphology consisting of nanoscale or submicron bainitic ferrite plates and retained austenite. The average width of the bainitic plate increased when the transformation temperature increased. The growing directions of bainitic plates or sheaves are marked by the colored dash line and circled numbers. For instance, the plates marked with ① and yellow dash lines parallel with each other, and the plates circled with the red dash line and marked ③ propagate in the same direction, as shown in Figure 2a. The paralleled bainitic sheaves indicated that the propagation is not random but follows specific directions. Propagation of the bainitic lathes or sheaves is in accordance with repeated nucleation and growth of the sub-units with a lenticular shape [19]. The sub-unit traces of the bainitic ferrite are separated by the films of austenite, as shown by the red dash circled areas in Figure 2b. The bainitic sheaves consist of plates that are composed of sub-units with the same growing direction. There are obvious angles between the sheaves with different growing directions, and the angles are the projections of the actual angles between the different bainitic ferrite sheaves on the section plane. There are some huge sub-units (high thickness) at the edges between the bainitic sheaves and austenite blocks at a high transformation temperature, as shown in Figure 2c,d. This is because the thickness of the sub-unit near the austenite blocks increases even after lengthening has ceased and plastic relaxation of austenite enables the sub-units to grow thicker [20]. In addition, the strength of the austenite at high temperature is not high enough to resist the thickness increase of the sub-units. The size of the sub-units is much larger at a higher temperature (for example at 350 °C), as shown in Figure 2d, and so the plates are thicker [21,22]. Meanwhile, the average length of the lath becomes shorter, as propagating is easily stopped by the adjacent stable austenite [23]. The edge line and contour surface can be clearly observed from the bainitic ferrite plates after etching and the sub-unit of the bainitic ferrite has a lenticular morphology, as shown in Figure 2d. Based on the above, one can draw the conclusion that the growth behavior and morphology of the bainitic sheaves depended on the scale and growth direction of bainite sub-unit.

### 3.2. Crystal Orientation Relationship

**Figure 3:**
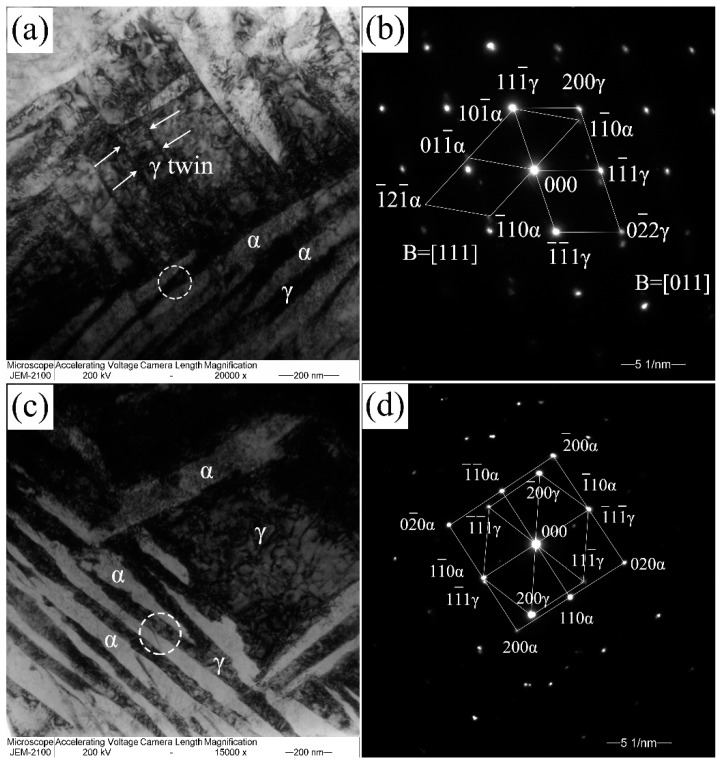
Transmission electron microscopy images of bainitic structure transformed at 200 °C (**a**) and 250 °C (**c**); the corresponding selected areas of electron diffraction (SAED) patterns are (**b**,**d**).

The bainitic transformation is considered a diffusionless transformation similar to the martensitic transformation. There is an assumption that the close-packed plane {110} of the bainitic ferrite is parallel to the {111} plane of the austenite, meaning that propagation of the bainite sheaves has specific orientations with the parent phase (austenite). The orientation relationship between the bainitic ferrite and austenite in 200 °C and 250 °C transformed coatings have a K-S relationship ({011}α//{111}γ, <111>α//<110>γ) and a N-W ({011}α//{111}γ, <112>α//<011>γ) relationship, as shown in Figure 3a,b, respectively. The difference between the K-S and N-W orientations is about 5.26° [24] and the deviation of the orientation relationship between bainitic ferrite and film austenite from K-S and N-W in the circled areas of Figure 3a,c are about 2.9° and 2.5°. It can be inferred that there is an orientation relationship of the nanostructured bainitic ferrite and adjacent austenite between the K-S and N-W relationships. These specific growth directions of the sheaves mentioned in Figure 2 were determined by the specific orientation relationships between bainitic ferrite and austenite.

**Figure 4:**
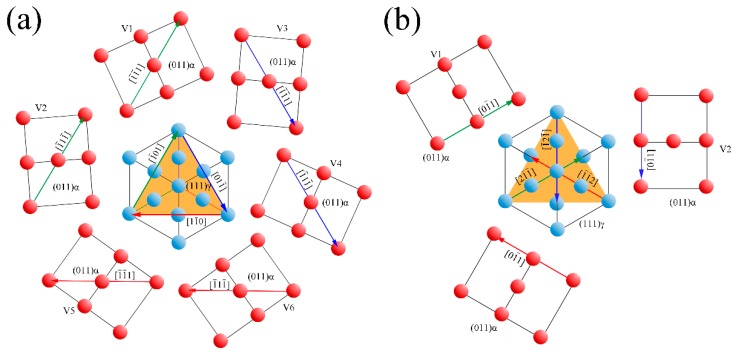
Illustration of the crystallographic variants evolving on the (111) austenite plane in the (**a**) K-S relationship and (**b**) N–W relationship.

Considering the symmetry of the cubic systems, there are crystallographic variants in the bainitic ferrite of the K-S and N-W orientation relationships evolving from a single grain of austenite that has specific growth orientations. The variants of the K-S and N-W relationships denoted as VX, where X indicates the variant number. Figure 4 shows six (V1–V6) K-S and three (V1–V3) N-W variants evolving on the (111) austenite plane. The orange triangle indicates the (111) austenite (fcc) plane and the rectangles show the (011) bainitic ferrite (bcc) plane. The color lines of the orange triangle and rectangles indicate the parallel directions of the austenite and bainite ferrite. Because there are four different {111} planes in austenite and six different <111> directions on these planes, the number of K-S variants is 24. Similarly, there are three different <112> directions on the {111} plane and the N-W variants number is 12.

**Figure 5:**
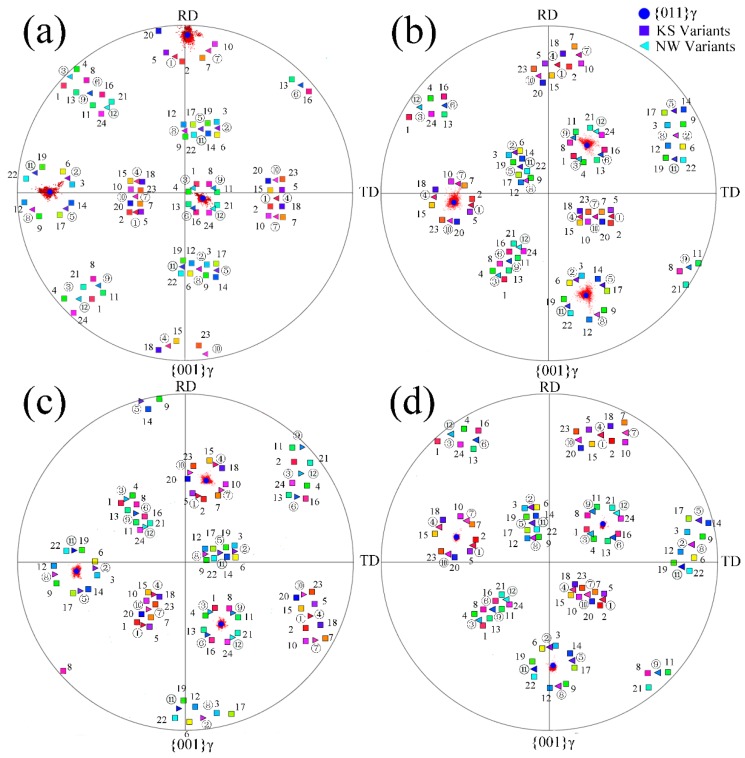
The {001} pole figures of the retained austenite in the (**a**) 200 °C; (**b**) 250 °C; (**c**) 300 °C and (**d**) 350 °C isothermal transformed microstructures, and the corresponding calculated K-S (colored squares) and N-W (colored triangles) variants of the bainitic ferrite.

The orientation relationship between the bainitic ferrite and austenite is investigated by TEM, and since the analytical area is quite small, EBSD is performed to characterize the phases of the fully transformed bainitic microstructures in a relatively large area. The orientation distribution of the {001} pole figures of the austenite in the area (30 μm × 30 μm) of the nano bainitic coatings is presented in Figure 5 (red scattered points). The orientation of the retained austenite in one single prior austenite grain at four isothermal transformation temperatures is determined and the average values are listed in Table 2. The 24 variants of the K-S and 12 variants of the N-W orientation relationships based on the orientation results in Figure 5 are calculated by the open source software (PTCLab), and the calculated results are listed in Table 3 and Table 4, respectively [25].

The 24 K-S variants and 12 N-W variants are calculated based on the orientation relationship of the prior austenite and the <100> directions shown in the {001} austenite pole figure. The color squares and triangles represent the K-S and N-W ideal relationships, respectively. The numbers of 1 to 24 and ① to ⑫ represent the serial number of the 24 K-S variants and 12 N-W variants of the bainitic ferrite, respectively, corresponding to the numbers in Table 3 and Table 4.

**Figure 6:**
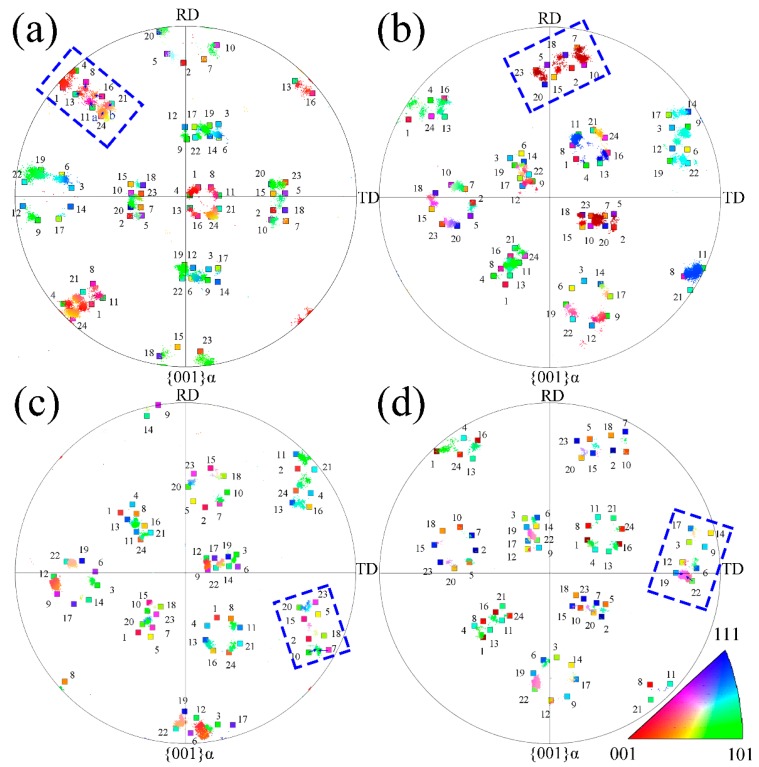
The determined {001} inverse pole figure of the bainitic ferrite and the comparison with the calculated variants of K-S relationship of the selected areas of microstructures transformed at (**a**) 200 °C; (**b**) 250 °C; (**c**) 300 °C and (**d**) 350 °C.

Figure 6 indicates the inverse pole figure (IPF) of the bainitic ferrite formed at different temperatures and the calculated K-S orientation relationship variants. The scattered points with different colors illustrate the orientation distributions of the transformed bainitic ferrite indicating that the actual orientation relationships of α and γ do not exactly match the K-S relationship when compared to the EBSD and calculated variants. The actual orientations of the bainitic ferrite deviate from the ideal calculated K-S orientation relationship. For instance, the small deviation between V24 to the actual orientation is a blue arrow, as shown in the blue dashed box in Figure 6a, from a to b. Not all 24 variants are observed from the pole figures. This is due to the fact that the interaction energy of each of the K-S variants is different when associated with the calculation result of the γ→α transformation (Kundu and Bhadeshia [26]). The number of variants in the same unit area decreases with increasing temperature, and hence the green, cyan, and purple regions illustrate that V1, V16, V19, and V20 are the dominant variants in the K-S orientation relationship of the 350 °C specimen. All the determined orientations of the bainitic ferrite deviate from the calculated K-S variants based on the average orientation of the retained austenite, as consistency with the TEM results and the deviation from the calculated orientations is more obvious at a high temperature.

**Figure 7:**
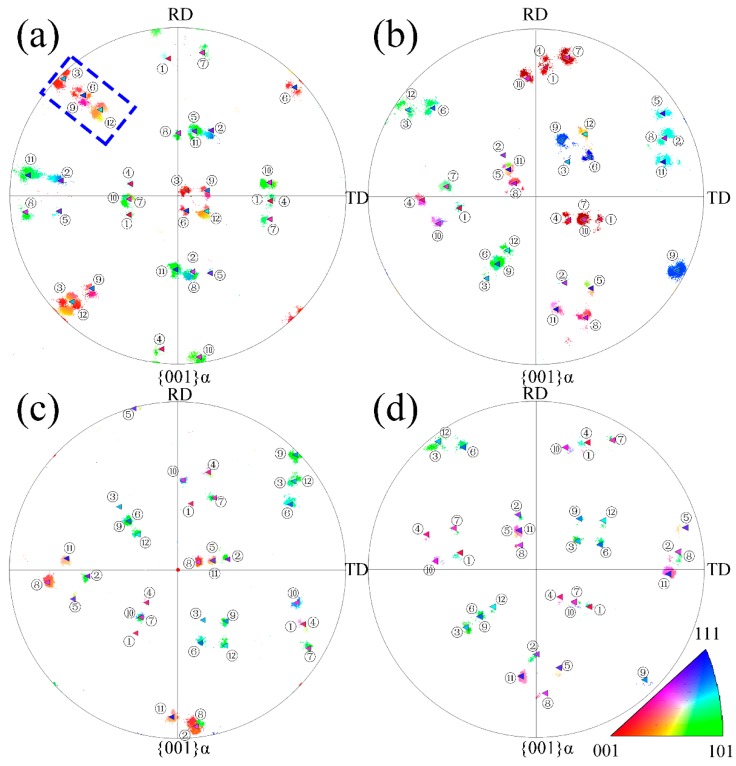
The determined {001} inverse pole figure of the bainitic ferrite and the comparison with the calculated variants of N-W relationship of the selected areas of microstructures transformed at (**a**) 200 °C; (**b**) 250 °C; (**c**) 300 °C and (**d**) 350 °C.

Figure 7 compares the bainitic ferrite orientations in the (001) inverse pole figure with the calculated results of the 12 variants of the N-W orientation relationship based on the average orientation of the retained austenite showing that the calculated N-W variants do not follow the inverse pole figures of the bainitic ferrite very well at a low temperature. For instance, the upper left of the inverse pole figure of the bainitic ferrite transformed at 200 °C is divided into eight parts, and the calculated N-W variants of the V3, V6, V9, and V12 are distributed between the two determined regions. The orientation distribution of bainitic ferrite is closer to the calculated results of the N-W variants as the isothermal temperature is increased. Therefore, the orientation of the bainitic ferrite corresponds to the 12 variants at 300 °C and 350 °C, as shown in Figure 7c,d, implying that the orientation relationship between the bainitic ferrite and austenite is closer to N-W than K-S. In addition, the orientation exhibits continuous gradients between the N-W and K-S as the isothermal transformation temperature is reduced.

**Figure 8:**
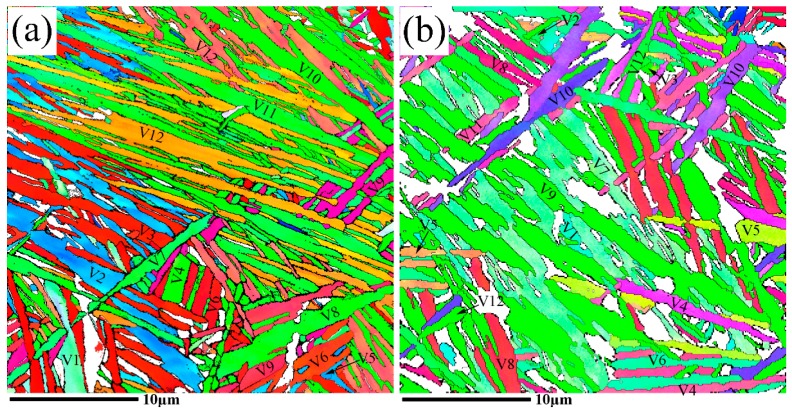

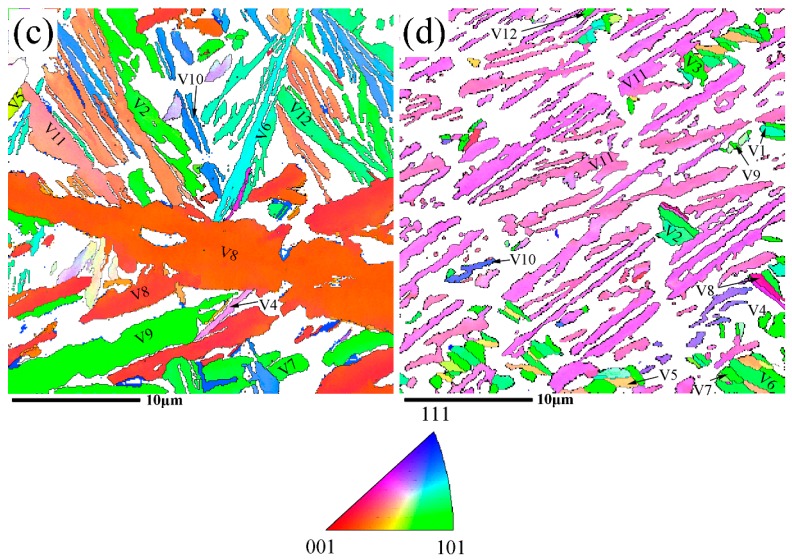
The IPF map of bainitic microstructure transformed at (**a**) 200 °C; (**b**) 250 °C; (**c**) 300 °C and (**d**) 350 °C.

Figure 8 presents the inverse pole figures of the nanobainitic microstructures transformed at different temperatures. The regions plotted with the same color represent the bainitic sheaves with the same growth orientations and the variant colors illustrate the different orientations of the bainitic ferrite corresponding to the color of the scattered regions in the inverse pole in Figure 6 and Figure 7. All 12 variants of the N-W relationship can be observed from the four figures and the bainitic lathes with different colors in the IPF map are numbered with the corresponding variants. The white regions in the microstructures are the retained austenite and the surface fraction of the retained austenite that goes up with increasing isothermal temperature. The variant selection is weakened at a low temperature because plastic relaxation of austenite is difficult. Hence, the variants of the bainitic ferrite becomes multiple as a result of the internal stress relaxation caused by the bainitic transformation misfit strain [27]. Consequently, there is no obvious dominant variant sheaf at 200 °C. V7, V8, V9, and V10 are dominant at 250 °C, whereas V8, V9, and V11 are dominant at 300 °C, and V11 in the N-W relationship is the only dominant one at 350 °C.

The orientation relationship of γ and α is between the K-S and N-W relationships at the low temperature of 200 °C. The bainitic ferrite is homogeneously distributed in the prior austenite grain. The orientation relationship becomes closer to the N-W relationship as the isothermal temperature is increased, and some sheaves with specific N-W variants are dominant in a given austenite grain. More retained austenite is obtained at a higher temperature.

**Figure 9:**
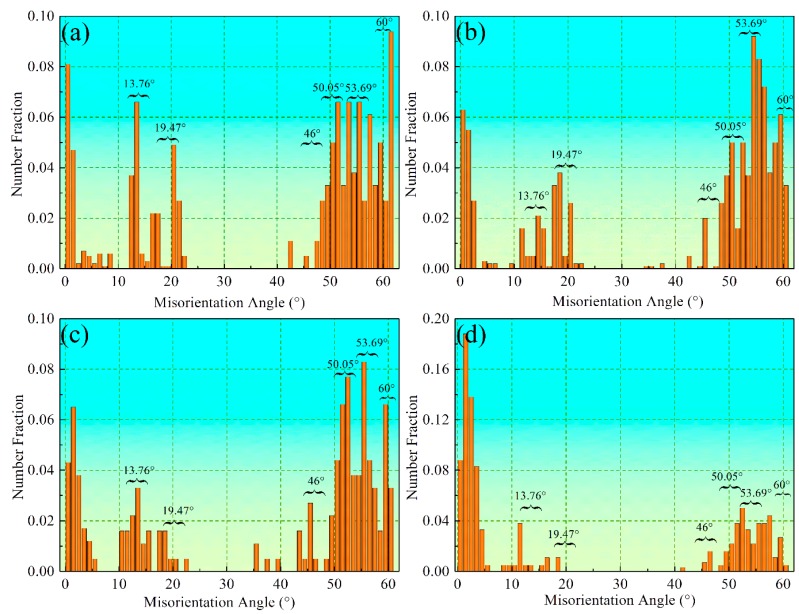
The misorientation angle/axis distribution for the given austenite grain transformed to bainite at different temperatures of (**a**) 200 °C; (**b**) 250 °C; (**c**) 300 °C and (**d**) 350 °C.

When the bainitic transformation occurs in a single austenite crystal (grain), there are 12 or 24 crystallographic variants in the N-W or K-S orientation relationships due to the symmetry of the cubic systems. There are five and ten possible misorientation angles between the different variants of the bainitic ferrite in the N-W relationship (13.76°, 19.47°, 50.05°, 53.69°, and 60°) and the K-S relationship (10.53°, 14.88°, 20.61°, 21.06°, 47.11°, 49.47°, 50.51°, 51.73°, 57.21°, and 60°), respectively [28], as shown in Table 3 and Table 4. The misorientation angles for a single austenite grain transformed to nanobainite at different isothermal temperatures are shown in Figure 9. The misorientation angles marked in the figures and 42.85° and 46° represent the interface of the bainitic ferrite and retained austenite that have the K-S or N-W relationship [14].

### 3.3. Variants of Growth Directions

**Figure 10:**
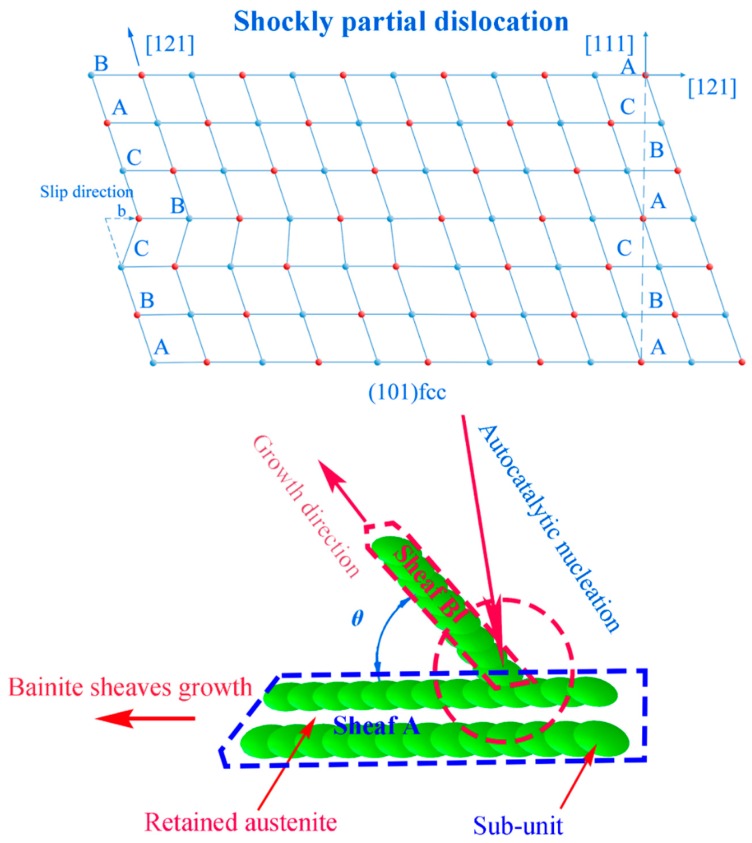
Schematic illustration describing the growth intersection angle of the initial bainitic lath and the lath caused by autocatalysis.

The misorientation of the growth direction initiates from nucleation of the new formed lath. The newly formed bainitic sub-unit nucleates at the phase boundary of the existing bainite and austenite and grows according to the N-W or K-S relationship with austenite, which is called autocatalysis (Figure 10). The initial bainite lath or sheaf propagates according to the mechanism of repeated nucleation and growth of the sub-units. When the new embryo of the bainite forms on defects such as dislocations, the new nucleus of bainitic ferrite grows at a misorientation angles (θ) with the prior propagation direction of the bainitic lath. The misorientation angles (θ) only have five possible values of 13.76°, 19.47°, 50.05°, 53.69°, and 60,° as discussed previously, and autocatalytic nucleation of the sub-unit is affected by the previously formed sub-units as a result of the strain of bainitic transformation [29]. In addition, nucleation occurs preferentially in the dislocations on the slip planes near the previously formed bainitic sub-units. The bainitic or martensitic transformation with the N-W relationship of the parent and product phases is related to partial dislocations [30] and the effect of the partial dislocation on autocatalysis is shown in the following:

(1) $$\frac{a}{2}\cdot [10\bar{1}]=\frac{a}{6}\cdot [2\bar{1}\bar{1}]+\frac{a}{6}[11\bar{2}]$$

**Figure 11:**
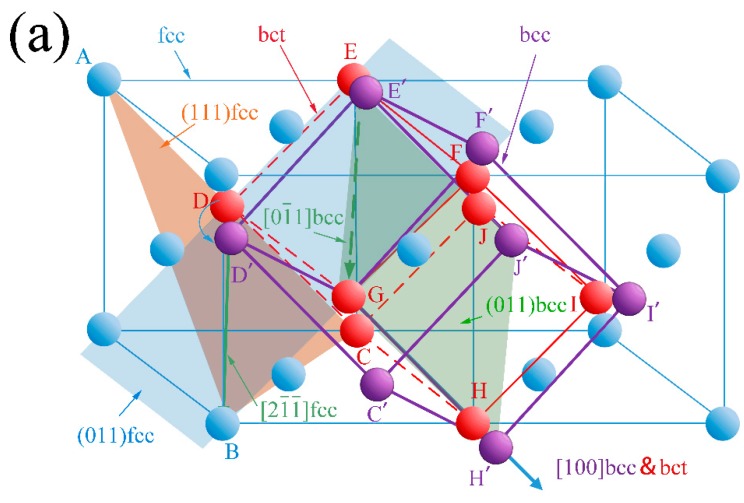

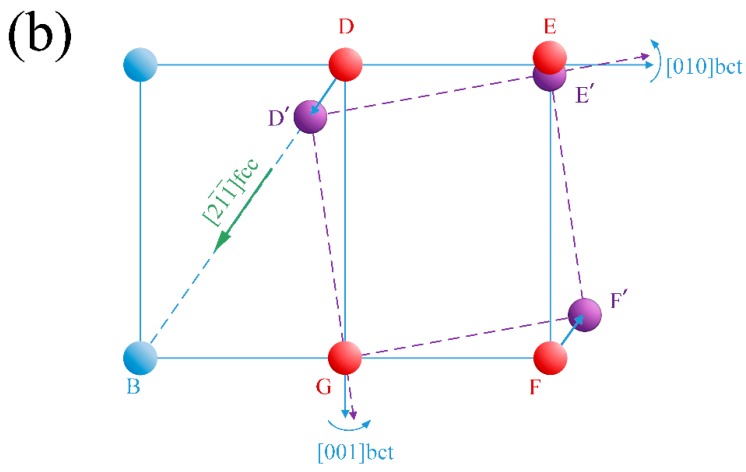
The lattice change during the bainitic transformation (fcc to bcc); (**a**) lattice rotation in V1 relationship, (111)γ//(111)α, [2$\bar{1}$$\bar{1}$]γ//[0$\bar{1}$1]α; (**b**) lattice distortion as Shockley partial dislocation mechanism on the (011) fcc plane.

The perfect dislocation $\frac{a}{2}$ [10$\bar{1}$] in the austenite plane (111) splits into two Shockley partial dislocations. The lattice change during the bainitic transformation process with V1 of the N-W relationship is shown in Figure 11.

The blue spheres and blue lines in Figure 11 indicate the fcc structure of the previous austenite and the purple spheres and lines indicate the bcc structure of bainitic ferrite. There is an assumed temporary bct structure in the fcc to bcc transformation, as shown by the red spheres and lines, and there is strain for the 9.7 ° rotation along the [100] axis. The triangle plotted as orange (ABC) shows the plane of (111) fcc which is paralleled to (011) bcc (plotted as a green parallelogram). The direction of [2$\bar{1}$$\bar{1}$] on the (111) plane of the fcc structure (DB) is parallel to the direction of [0$\bar{1}$1] on the (011) plane of the bcc structure ($E^{\prime }$G). The lattice of the austenite is distorted and displaced along the Burgers vector of the Shockley partial dislocation. The lattice displaces the plane of (011) fcc when atom D moves to D’, E moves to E’, and F moves to F’, as shown in Figure 11b. It is obvious that the bct lattice should change to the bcc lattice by contraction of the [001] axis and extension of the [010] axis, thereby making lattice distortion happen easily through V1 of the NW relationship.

There are 24 equivalent Burgers vectors in the austenite slip system dissociated to 48 equivalent Shockley partial dislocations. Considering the symmetry of the cubic structures, there are 12 variants of the N-W orientation relationship between the austenite and bainitic ferrite. Apparently, the bainitic microstructure propagates with the specific variants orientation from one original austenite grain, and this is also the reason why there are sheaves with different growth directions, as shown in Figure 2.

## 4. Conclusions

The orientation relationship of bainitic ferrite and austenite does not follow the ideal K-S and N-W relationship. Rather, the orientation relationship falls between the ideal K-S and N-W. The orientation relationship is closer to the N-W relationship at a high transformation temperature and the mixed N-W and K-S relationships are observed as the transformation temperature decreases. The propagation directions of the bainitic sheaves are dominated by specific variants as the temperature increases. The phenomenon stems from decomposition of the dislocation structures during repeated autocatalytic nucleation during transformation. The variants of the propagation directions of the bainitic sheaves are caused by Shockley partial dislocations and the smaller dislocation densities cause enhanced variant selection of the nanobainitic microstructures at high isothermal temperature. The morphology of the bainitic ferrite lath changes from long-and-slender to short-and-thicker as the transformation temperature decreases. A smaller lengthening of the bainitic lathes is caused by propagation halted by adjacent stable austenite and the larger thickness results from the lower strength of the primary austenite to resist thickening of the bainitic sub-units. The edge line of the bainitic lath provides evidence that the morphology of the sub-unit has a lenticular shape and the growth follows the pile-up mechanism.

## Figures and Tables

**Table 1:** Processing parameters for laser cladding and isothermal transformation.

Laser Cladding				Isothermal Heat Treatment	
Laser Power (W)	Preheating Temperature (°C)	Scanning Velocity (mm/s)	Powder Feed Rate (g/min)	Isothermal Temperature (°C)	Isothermal Times (h)
2500	220	10	30	200	1, 2, 4, 8, 12, 24, 48,
2400	270	10	30	250	1/2, 1, 2, 3, 4, 8, 12, 16
2300	320	10	30	300	1/4, 1/2, 3/4, 1, 3/2, 2, 3, 4, 6
2200	370	10	30	350	1/4, 1/3, 1/2, 3/4, 1, 1.5, 2, 3, 4

**Table 2:** The orientation of the retained austenite of the selected area at different temperatures.

Isothermal Temperature (°C)	Orientation of the Retained Austenite	
	Plane	Direction
200	(0.0898 0.2007 0.9755)	[0.9955 0.0114 −0.094]
250	(0.5175 0.4221 0.7443)	[−0.0624 −0.8489 0.5248]
300	(0.6015 0.4011 0.6909)	[0.7738 −0.0776 −0.6286]
350	(0.5122 0.4406 0.7373)	[0.2266 −0.8973 0.3788]

**Table 3:** The 24 variants for the K-S orientation relationship (V1–V24).

Variant	Parallel Plane	Parallel Direction	Rotation Axis in V1	Rotation Angle (deg)
V1	(111)γ//(011)α	[$\bar{1}$01]γ//[$\bar{1}$$\bar{1}$1]α		
V2	(111)γ//(011)α	[$\bar{1}$01]γ//[$\bar{1}$1$\bar{1}$]α	[0.58 −0.58 0.58]	60.00
V3	(111)γ//(011)α	[01$\bar{1}$]γ//[$\bar{1}$$\bar{1}$1]α	[0.00 −0.71 −0.71]	60.00
V4	(111)γ//(011)α	[01$\bar{1}$]γ//[$\bar{1}$1$\bar{1}$]α	[0.00 0.71 0.71]	10.53
V5	(111)γ//(011)α	[1$\bar{1}$0]γ//[$\bar{1}$$\bar{1}$1]α	[0.00 0.71 0.71]	60.00
V6	(111)γ//(011)α	[1$\bar{1}$0]γ//[$\bar{1}$1$\bar{1}$]α	[0.00 −0.71 −0.71]	49.47
V7	(1$\bar{1}$1)γ//(011)α	[10$\bar{1}$]γ//[$\bar{1}$$\bar{1}$1]α	[−0.58 −0.58 0.58]	49.47
V8	(1$\bar{1}$1)γ//(011)α	[10$\bar{1}$]γ//[$\bar{1}$1$\bar{1}$]α	[0.58 −0.58 0.58]	10.53
V9	(1$\bar{1}$1)γ//(011)α	[$\bar{1}$$\bar{1}$0]γ//[$\bar{1}$$\bar{1}$1]α	[−0.19 0.77 0.61]	50.51
V10	(1$\bar{1}$1)γ//(011)α	[$\bar{1}$$\bar{1}$0]γ//[$\bar{1}$1$\bar{1}$]α	[−0.49 −0.46 0.74]	50.51
V11	(1$\bar{1}$1)γ//(011)α	[011]γ//[$\bar{1}$$\bar{1}$1]α	[0.35 −0.93 −0.07]	14.88
V12	(1$\bar{1}$1)γ//(011)α	[011]γ//[$\bar{1}$1$\bar{1}$]α	[0.36 −0.71 0.60]	57.21
V13	($\bar{1}$11)γ//(011)α	[0$\bar{1}$1]γ//[$\bar{1}$$\bar{1}$1]α	[0.93 0.35 0.07]	14.88
V14	($\bar{1}$11)γ//(011)α	[0$\bar{1}$1]γ//[$\bar{1}$1$\bar{1}$]α	[0.74 0.46 −0.49]	50.51
V15	($\bar{1}$11)γ//(011)α	[$\bar{1}$0$\bar{1}$]γ//[$\bar{1}$$\bar{1}$1]α	[−0.25 −0.63 −0.74]	57.21
V16	($\bar{1}$11)γ//(011)α	[$\bar{1}$0$\bar{1}$]γ//[$\bar{1}$1$\bar{1}$]α	[0.66 0.66 0.36]	20.61
V17	($\bar{1}$11)γ//(011)α	[110]γ//[$\bar{1}$$\bar{1}$1]α	[−0.66 0.36 −0.66]	51.73
V18	($\bar{1}$11)γ//(011)α	[110]γ//[$\bar{1}$1$\bar{1}$]α	[−0.30 −0.63 −0.72]	47.11
V19	(11$\bar{1}$)γ//(011)α	[$\bar{1}$10]γ//[$\bar{1}$$\bar{1}$1]α	[−0.61 0.19 −0.77]	50.51
V20	(11$\bar{1}$)γ//(011)α	[$\bar{1}$10]γ//[$\bar{1}$1$\bar{1}$]α	[−0.36 −0.60 −0.71]	57.21
V21	(11$\bar{1}$)γ//(011)α	[0$\bar{1}$$\bar{1}$]γ//[$\bar{1}$$\bar{1}$1]α	[0.96 0.00 −0.30]	20.61
V22	(11$\bar{1}$)γ//(011)α	[0$\bar{1}$$\bar{1}$]γ//[$\bar{1}$1$\bar{1}$]α	[−0.72 0.30 −0.63]	47.11
V23	(11$\bar{1}$)γ//(011)α	[101]γ//[$\bar{1}$$\bar{1}$1]α	[−0.74 −0.25 0.63]	57.21
V24	(11$\bar{1}$)γ//(011)α	[101]γ//[$\bar{1}$1$\bar{1}$]α	[0.91 −0.41 0.00]	21.06

**Table 4:** The 12 variants for the N-W orientation relationship (V1–V12).

Variant	Parallel Plane	Parallel Direction	Rotation Axis in V1	Rotation Angle (deg)
V1	(111)γ//(011)α	[2$\bar{1}$$\bar{1}$]γ//[0$\bar{1}$1]α		
V2	(111)γ//(011)α	[$\bar{1}$21]γ//[0$\bar{1}$1]α	[0.000 −0.707 −0.707]	60.00
V3	(111)γ//(011)α	[$\bar{1}$$\bar{1}$2]γ//[0$\bar{1}$1]α	[0.00 0.707 0.707]	60.00
V4	($\bar{1}$11)γ//(011)α	[$\bar{2}$$\bar{1}$$\bar{1}$]γ//[0$\bar{1}$1]α	[1.000 0.000 0.000]	49.47
V5	($\bar{1}$11)γ//(011)α	[12$\bar{1}$]γ//[0$\bar{1}$1]α	[−0.223 −0.697 −0.681]	10.53
V6	($\bar{1}$11)γ//(011)α	[0$\bar{1}$2]γ//[0$\bar{1}$1]α	[−0.223 0.697 0.681]	50.51
V7	(1$\bar{1}$1)γ//(011)α	[21$\bar{1}$]γ//[0$\bar{1}$1]α	[0.706 −0.706 −0.060]	14.88
V8	(1$\bar{1}$1)γ//(011)α	[$\bar{1}$$\bar{2}$$\bar{1}$]γ//[0$\bar{1}$1]α	[−0.681 −0.223 0.697]	50.51
V9	(1$\bar{1}$1)γ//(011)α	[$\bar{1}$12]γ//[0$\bar{1}$1]α	[−0.624 0.471 −0.624]	57.21
V10	($\bar{1}$$\bar{1}$1)γ//(011)α	[2$\bar{1}$1]γ//[0$\bar{1}$1]α	[0.706 0.706 0.060]	50.51
V11	($\bar{1}$$\bar{1}$1)γ//(011)α	[$\bar{1}$21]γ//[0$\bar{1}$1]α	[−0.624 −0.471 0.624]	57.21
V12	($\bar{1}$$\bar{1}$1)γ//(011)α	[$\bar{1}$$\bar{1}$2]γ//[0$\bar{1}$1]α	[−0.681 0.223 −0.697]	20.61
